# Area-Efficient Post-Processing Circuits for Physically Unclonable Function with 2-Mpixel CMOS Image Sensor

**DOI:** 10.3390/s21186079

**Published:** 2021-09-10

**Authors:** Shunsuke Okura, Masanori Aoki, Tatsuya Oyama, Masayoshi Shirahata, Takeshi Fujino, Kenichiro Ishikawa, Isao Takayanagi

**Affiliations:** 1 Department of Science and Engineering, Ritsumeikan University, 1-1-1 Noji-higashi, Kusatsu 525-8577, Shiga, Japan; ri0076kk@ed.ritsumei.ac.jp (M.A.); fujino@se.ritsumei.ac.jp (T.F.); 2 Graduate School of Science and Engineering, Ritsumeikan University, 1-1-1 Noji-higashi, Kusatsu 525-8577, Shiga, Japan; ri0068hi@ed.ritsumei.ac.jp; 3 Research Organization of Science and Technology, Ritsumeikan University, 1-1-1 Noji-higashi, Kusatsu 525-8577, Shiga, Japan; msr10001@fc.ritsumei.ac.jp; 4 Brillnics Japan Inc., 6-21-12 Minami-Oi, Shinagawa-ku, Tokyo 140-0013, Japan; ishikawa.kenichiro@brillnics.com (K.I.); takayanagi.isao@brillnics.com (I.T.)

**Keywords:** imaging, integrated circuit reliability, CMOS image sensors, IoT, hardware security, PUF, device authentication

## Abstract

In order to realize image information security starting from the data source, challenge–response (CR) device authentication, based on a Physically Unclonable Function (PUF) with a 2 Mpixel CMOS image sensor (CIS), is studied, in which variation of the transistor in the pixel array is utilized. As each CR pair can be used only once to make the CIS PUF resistant to the modeling attack, CR authentication with CIS can be carried out 4050 times, with basic post-processing to generate the PUF ID. If a larger number of authentications is required, advanced post-processing using Lehmer encoding can be utilized to carry out authentication 14,858 times. According to the PUF performance evaluation, the authentication error rate is less than 0.001 ppm. Furthermore, the area overhead of the CIS chip for the basic and advanced post-processing is only 1% and 2%, respectively, based on a Verilog HDL model circuit design.

## 1. Introduction

As the Internet of Things (IoT) develops, a vast number of sensors are expected to become connected to the internet [1], in order to collect a huge amount of data. Security in IoT devices is of paramount importance for the further development of related technology [2,3]. To achieve sufficient information security, data confidentiality, data integrity, and device authentication are required. For such functions, a Physically Unclonable Function (PUF) [4] can serve as a unique identifier (ID) and key for a device, based on physical variations caused during the manufacturing process [5,6,7,8,9].

The strong dependence on the internal parameters makes a PUF a highly tamper-evident ID, providing key storage without non-volatile memory (NVM). Therefore, a PUF can provide security that starts at the data source, in order to prevent attackers from exploiting sensor networks.

One challenge of PUF is the instability caused by random noise and temporary changes in the PUF ID bits. In order to make the PUF ID reliable, error correction [10] or masking of unstable bits [11] are utilized. However, the sensor devices, which process analog signals, are not fabricated with scaled-down CMOS technologies, and the processing capability is limited compared to digital devices such as FPGA, microcontrollers, CPU, and so on. Therefore, a more lightweight function is required for the sensor devices by eliminating the use of error correction circuits. Another method to make the PUF reliable is hot carrier injection (HCI) burn-in [12], in which the physical variation is enlarged. However, the HCI burn-in degrades the sensor performance due to the enlarged physical variation and is difficult to apply to the sensor PUF device. For these reasons, device authentication based on the fuzzy identification scheme with a threshold is preferred for the sensor PUF. In the fuzzy identification, a PUF device is authenticated when the the temporal change is less than the set threshold.

**Figure 1:**
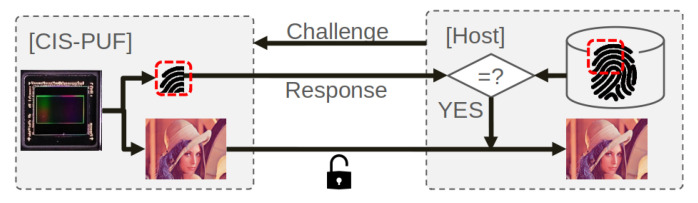
Verification phase of challenge–response authentication.

For image information security, a CMOS image sensor (CIS) with a PUF has been proposed [9,13], in which variation of a transistor that is conventionally implemented in the pixel array is utilized as the source of the PUF ID. The advantage of the these CIS PUFs is the small circuit overhead because the pixel transistor is utilized to readout the photo conversion electron integrated in the pixel during an imaging mode, removing the transistor variation. CIS PUF-based device authentication is realized by a challenge–response (CR) authentication scheme, which consists of two phases: enrollment and verification [14]. During the enrollment phase, all the PUF ID bits derived from the pixel array are recorded by the verifier. During the verification phase, shown in Figure 1, the verifier issues a challenge, consisting of a randomly selected pixel address. The CIS must respond with the one PUF ID string that fits the challenge the verifier issued. A CIS device is authenticated when the Hamming distance (HD) of the regenerated response and the enrolled response is less than a set threshold. The verifier issues a different challenge each time; thus, knowing previous CR pairs is of no use. Even though this one-time scheme is utilized to avoid replay attacks, the number of authentications is limited. Because of this simple scheme, the device authentication comprises a lightweight security function and requires small circuit overhead in the CIS device.

In this paper, device authentication using the CIS is studied, as the processing capability of the considered CIS device is limited. However, the available number of times of device authentication is limited due to the one-time scheme. The extensive PUF, in which the PUF response is generated from random pairs of the pixel transistor, has large space of CR pairs [9] but is vulnerable to modeling attacks with machine learning [15,16] since multiple responses are generated based on the same pixels. On the other hand, the confined PUF, in which the PUF response is generated from fixed pairs of the pixel transistor, has a small space of CR pairs [13]. For the case in which a larger CR pair space is required, the Lehmer encoding is applied to the confined PUF and the PUF performance is evaluated with measurement data. Furthermore, the post-processing circuit to derive the PUF ID from the transistor variation is presented in order to estimate the circuit overhead for the CIS chip.

Section 2 provides an overview of the CIS PUF and the post-processing circuits to generate the PUF ID, followed by the evaluation result of the PUF performance, shown in Section 3. In Section 4, the design example with the Verilog HDL model is described. Section 5 summarizes this paper.

## 2. Overview of CIS PUF

The CIS PUF utilizes the variation of a transistor implemented in the pixel array as the source of the PUF ID in PUF mode. Once the CIS is switched to an imaging mode (in accordance with the control register setting), the PUF ID cannot be easily copied, as the transistor variation is removed in order to capture an image with small pixel-to-pixel fixed pattern noise.

### 2.1. Circuits and Operations

**Figure 2:**
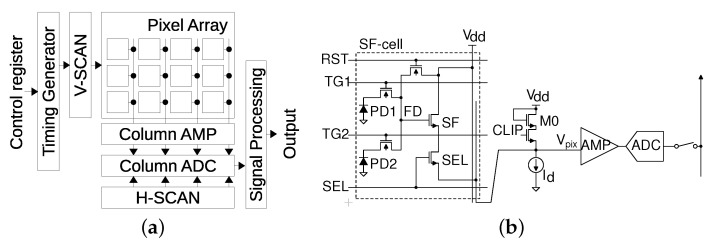
Block diagram of the CIS and a column readout circuit. (**a**) Chip overview. (**b**) Column readout circuit.

Figure 2 shows a chip overview of the CIS and a column readout circuit [13].

The pixel array is composed of 2 Mpixels using a 2-shared pixel structure; note that the shared pixel is called an SF cell in this paper. A timing generator controls the vertical scanner, V-SCAN, which drives the pixels row-by-row. In accordance with the control register, the chip operation mode is switched between the imaging mode and the PUF mode. The pixel output voltage is converted into a 12-bit signal with the column readout circuit. The digitized pixel signal is then transferred to a signal processing circuit pixel-by-pixel by the horizontal scanner, H-SCAN. The clip transistor (M0) is used to reduce the Vdd/ground bounce during the imaging mode, as well as to derive the threshold voltage of the SF transistor ($V_{th,SF}$) in the SF cell during the PUF mode. As the pixel that processes analog signals is not fabricated with an advanced fine CMOS process, the $V_{th,SF}$ mismatch exceeds the millivolt range, which is larger than the readout circuit noise.

**Figure 3:**
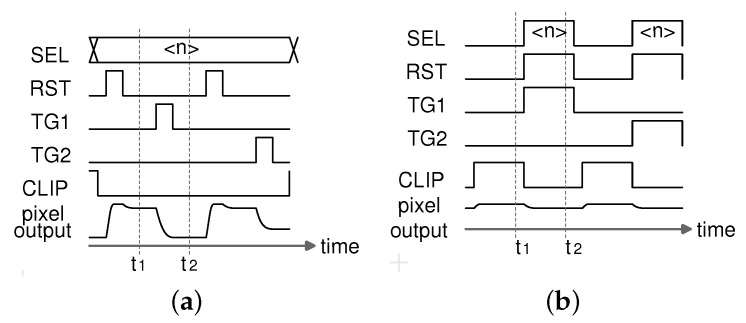
Timing diagrams of imaging mode and PUF mode. (**a**) Imaging mode. (**b**) PUF mode.

The timing diagrams are shown in Figure 3. During the imaging mode shown in Figure 3a, the pixels in the *n*th row are reset setting RST high, and the pixel output voltage is given by

(1) $$\begin{array}{c} V_{pix}\left(t_{1}\right)=V_{dd}-V_{th,SF}-\Delta_{ov,SF}, \end{array}$$

where $\Delta_{ov,SF}$ is the overdrive voltage of the SF transistor. The signal voltage is then read out and the pixel output voltage is given by

(2) $$\begin{array}{c} V_{pix}\left(t_{2}\right)=V_{dd}-V_{th,SF}-\Delta_{ov,SF}-\frac{q_{sig}}{C_{FD}}, \end{array}$$

where $C_{FD}$ and $q_{sig}$ are the capacitance on the node FD and the photo electron charge transferred from PD1 to the FD capacitor during high TG, respectively.

The column amplifier removes $V_{th,SF}$ and derives $q_{sig}$ through the subtraction of the reset and signal voltages, as follows:

(3) $$\begin{array}{c} V_{pix}\left(t_{1}\right)-V_{pix}\left(t_{2}\right)=\frac{q_{sig}}{C_{FD}}. \end{array}$$

On the other hand, during the PUF mode shown in Figure 3b, the difference in the output voltages of a clip-transistor M0 and an SF transistor in the *n*th row is obtained from the readout signals at $t_{1}$ and $t_{2}$. The differential output, $V_{PUF}$, is the source of the PUF ID. The operation is given as follows:

(4)Vpix(t1)=Vdd−Vth,M0−Δov,M0,(5)Vpix(t2)=Vdd−Vth,SF−Δov,SF,VPUF≡Vpix(t1)−Vpix(t2)=Vth,SF−Vth,M0+Δov,SF−Δov,CLIP(6)≈Vth,SF−Vth,M0,

where the variation of $\Delta_{ov}$, which is smaller than that of $V_{th}$, is ignored for simplicity. The photo electron charge integrated in the PD1, $q_{sig}$, is removed by setting RST and TG high simultaneously. Thus, transistor threshold voltages are dominant even under light exposure. Even though the variation of $V_{th,SF}$ is independent and identically distributed in the SF cell array, the variation of $V_{th,M0}$ is common in a column, which results in column fixed pattern noise (FPN). The column FPN, which degrades the uniqueness of the PUF ID, should be removed.

### 2.2. Signal Processing to Generate PUF Response

In order to reduce the column FPN, we compared vertically adjacent cells to generate PUF responses for basic signal processing [13]. Furthermore, if a larger number of PUF responses is required, Lehmer and Gray encoding [17] can be applied as advanced L.G. signal processing. Even though the extensive PUF scheme, in which two randomly selected cells are compared [9], can yield a huge number of PUF responses, the response is vulnerable to modeling attack, as multiple responses are generated based on the same SF transistor [16]. Thus, the random pair comparison is not considered in this paper.

#### 2.2.1. Basic Signal Processing

**Figure 4:**
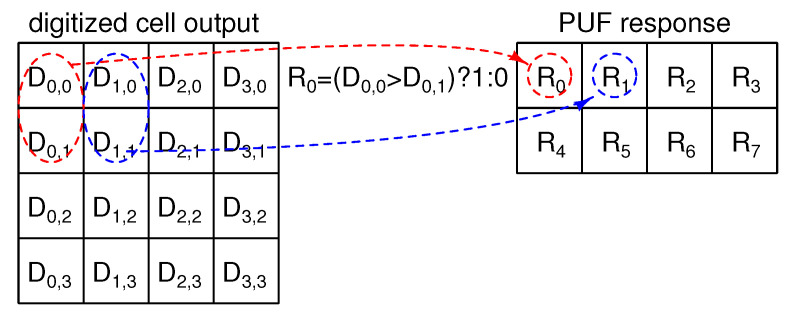
Operation diagram of basic signal processing to generate PUF response.

As basic signal processing, a PUF response bit is generated by comparing the two vertically adjacent SF cell outputs shown in Figure 4, where $D_{x,y}$ is the 12-bit digitized $V_{PUF}$ of the SF cell at address (x,y) and the number of SF cells is 16, for simplicity. The response $Q_{0}$ is 1 if $D_{0,0}>D_{0,1}$, and 0 otherwise. The binarized PUF response, *R*, is robust to variations in the environmental conditions, such as voltage and temperature, as an environmental change will affect all SF cells in a similar way. The comparison of two vertically adjacent cells can remove the column FPN caused by $V_{th,M0}$, resulting in almost ideal entropy [18]. The total number of PUF response bits generated from 16 SF cells is 8 bits.

For the 2-Mpixel CIS using a 2-shared pixel structure, shown in Figure 2, the total number of PUF response bits is 518.4 kbit as the total number of SF cells in the array is $1036.8\times 10^{3}\left(=1920\times 1080/2\right)$. Suppose that an 128-bit length response is utilized as a device ID for authentication. The available number of device authentications, in order to avoid spoofing attacks, is 4050(=518.4k/128), which is nearly equivalent to an authentication per day for 10 years. For some applications, this might not be enough and, so, Lehmer encoding [19] is further discussed to increase the number of CR pairs.

#### 2.2.2. L.G. Signal Processing

A combination of Lehmer encoding and Gray encoding, which is called L.G. encoding, is utilized to increase the number of CR pairs of oscillation-based PUFs [17,20]. The Lehmer code of the ascending order of the *n* oscillator outputs can take n! possible values and yield an effective $\log_{2}n!$-bit PUF response. The total PUF response bit is 5 bits if n=4, as the effective number of PUF response bits is 4.58 bits. A binary Gray encoding is then used to decrease the bit unreliability in case the order varies, as the Gray code guarantees that consecutive numbers only differ by one bit. The Lehmer encoding is applicable for the ring oscillator (RO) PUF, in which the RO output is an analog signal and can be sorted. Similarly, the Lehmer encoding is applicable to the CIS PUF because the $V_{th,SF}$ is read out with 12-bit ADC, similarly to the image signal.

**Figure 5:**
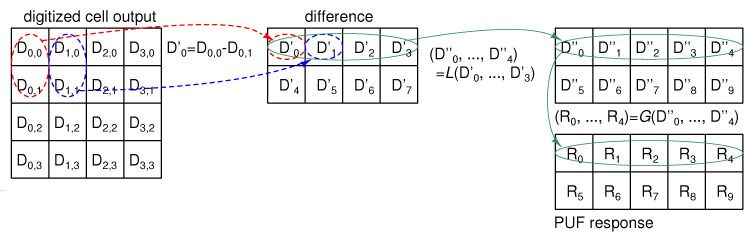
Operation diagram of L.G. post-processing.

Figure 5 shows the presented L.G. post-processing for the CIS-PUF. First, the column FPN is removed using the vertical difference in the SF cell output $\left(D_{0}^{\prime }=D_{0,0}-D_{0,1}\right)$. The Lehmer codes $D_{0}^{\prime \prime }$ and $D_{1}^{\prime \prime }$ count the number of terms in $\left(D_{1}^{\prime },D_{2}^{\prime },D_{3}^{\prime }\right)$ that are smaller than $D_{0}^{\prime }$, and the possible number of combinations is 00, 01, 10, and 11. The codes $D_{2}^{\prime \prime }$ and $D_{3}^{\prime \prime }$ count the number of terms in $\left(D_{2}^{\prime },D_{3}^{\prime }\right)$ that are smaller than $D_{1}^{\prime }$, and the possible numbers of combinations are 00, 01, and 10. Similarly, $D_{4}^{\prime \prime }$ is 0 or 1. As $D_{2}^{\prime \prime }$ and $D_{3}^{\prime \prime }$ include the invalid code 11 and does not have effective 2-bit information, the total effective number of bits is 4.58 bits. Using a binary Gray encoding for the Lehmer code output makes the overall response bit *R* noise-resilient. The total number of PUF response bits generated from the 16 SF cell outputs is 10 bits, which is 25% larger than that when using the basic post-processing.

For the 2-Mpixel CIS with L.G. encoding at n=32, the total number of effective PUF response bits is 1902 kbit $\left(=1920\times 270\times \log_{2}32!/32\right)$. The available number of authentications is 14,858 times, which is equivalent to 4 times authentication per day for 10 years.

### 2.3. Operation Confirmation of Device Authentication

**Figure 6:**
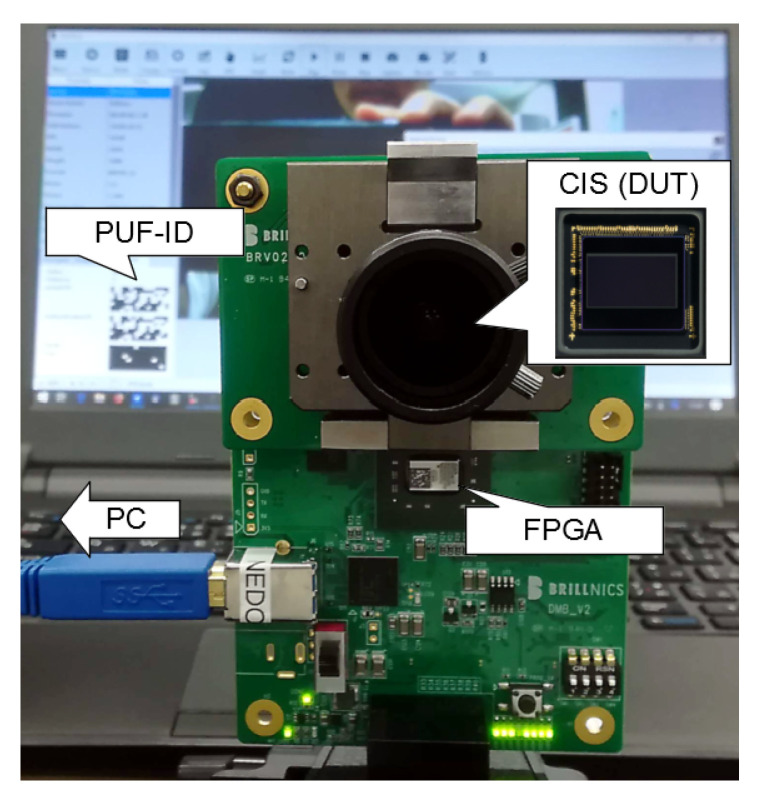
Operation confirmation setup for device authentication with CIS PUF.

Device authentication with CIS PUF was confirmed with the environment shown in Figure 6. The Brillnics BRV0200, which employs a 2 Mpixel CIS using a 2-shared pixel structure, was mounted on the camera board. A command was sent from the computer PC to the FPGA over USB, and was forwarded to the CIS chip over I2C in order to set the CIS chip to the PUF mode. The SF transistor variation was read out from the CIS device to the FPGA over a mobile industry processor interface (MIPI) camera serial interface (CSI), was transferred to the PC over USB, and was then post-processed in the PC. The PC identified the CIS chip by verifying the regenerated PUF response with the enrolled PUF response. When the regenerated PUF response was close to the enrolled one, the PC sent a command to set the CIS to the imaging mode as the authentication was successful, and started the communication of image data.

## 3. Evaluation of PUF Properties

The PUF-ID is evaluated using Brillnics BRV0200, which employs a 2—Mpixel 12-bit CMOS image sensor. The variation of the SF cell array is derived from 18 CIS chips and then post-processed offline in a computer.

**Figure 7:**
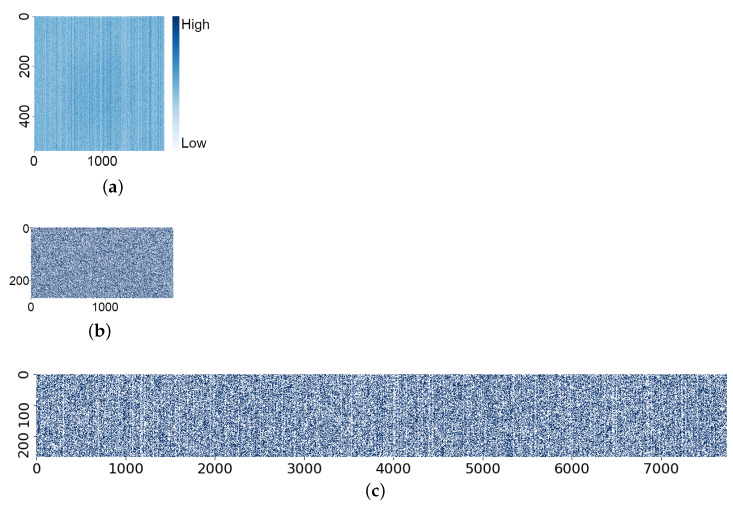
An example of SF cell and PUF response. (**a**) Heat-map diagram of SF cell output. (**b**) Heat-map diagram of basic PUF response. (**c**) Heat-map diagram of LG32 PUF response.

Figure 7 shows the measurement results for a sample CIS PUF chip.

The SF cell variation without post-processing ($V_{PUF}$), as shown in Figure 7a, includes considerable column FPN, in which the vertical and horizontal axis are, respectively, consistent with respect to the X and Y address of the SF cell in the 1920×540 array. Vertical stripes caused by the column FPN are visible, where the standard deviation among the columns is 58% of that among the SF cells. As the $V_{PUF}$ of an SF cell in a “low” or “high” column can be predicted from another SF cell output in the same column, the basic post-processing reduces the column FPN in the PUF response, as shown in Figure 7b. Figure 7b shows that the PUF response is randomly distributed compared to the SF cell output distribution shown in Figure 7a. The minimum entropy of the PUF response bit with the vertical column FPN reduction is over 0.99, while the minimum entropy without the vertical column FPN reduction is only around 0.03 [18]. The PUF response with L.G. post-processing is shown in Figure 7c, in which the range of the horizontal axis is extended with the Lehmer encoding.

The CIS PUF performance was quantitatively evaluated, regarding its reliability and uniqueness, and the device authentication error rate was estimated.

### 3.1. Reliability and Uniqueness

In order to quantitatively evaluate the reliability and the uniqueness of the generated PUF response, an intra-Hamming distance and an inter-Hamming distance were utilized [21]. The Hamming distance (HD) is the number of bits in which corresponding bit data differ.

The intra-HD evaluated with multiple outputs of a given device represents the reliability. In particular, 100 frames of SF cell array data were captured from a given CIS chip and then post-processed to derive PUF response data. Two of 100 frames of the array data were then compared to each other. The bit difference among the compared frame data is caused by random noise. As long as the noise is zero, the intra-HD is 0 bits and the same PUF ID is always repeated. The intra-HD was evaluated with the 128 bit PUF response cutout from the 2-Mpixel CIS. The total number of PUF ID samples with 18 CIS chips was therefore $3.6\times 10^{8}\left(=\frac{100!}{2!\times 98!}\times 18\times 4050\right)$.

The inter-HD evaluated with outputs of multiple devices represents the uniqueness. In particular, 18 chips of SF cell array data were captured, and then 2 of 18 chips of the array data were compared to each other. The bit difference among the compared chip data is caused by device-to-device mismatches. As long as the mismatch is non-correlated, the inter-HD shows a normal distribution, where the mean is 50% of bits and the PUF ID is different to the others among the devices. The inter-HD was evaluated with 128 bit PUF response cutout from the 2-Mpixel CIS. The total number of PUF ID samples with 100 frames per chip is therefore $6.2\times 10^{7}\left(=100\times \frac{18!}{2!\times 16!}\times 4050\right)$.

**Figure 8:**
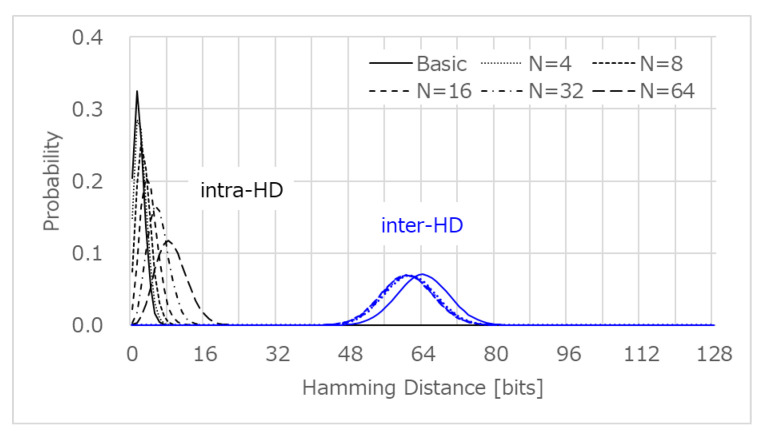
Measurement results of intra-HD and inter-HD.

Figure 8 shows the intra-HD (reliability) and inter-HD (uniqueness) of the PUF ID, in which the PUF ID is composed of a 128-bit PUF response. The average (μ) and standard deviation (σ) of intra-HD with the basic post-processing were only 1.58 and 1.25 bits, respectively, which means that the 123-bit PUF response does not change among the enrollment and verification, with a probability of over 99.7% (3σ) in the device authentication process. The reliability was very high as the variation of $V_{th,SF}$ was larger than that of random noise. The intra-HD under the temperature and voltage variation was also small [13], because the power supply voltage was canceled, as shown in Equation (6), and the $V_{th,SF}$ will shift in a similar way. As *n* increases with the L.G. encoding, the intra-HD increased and reliability decreased, due to the higher probability of the comparison of two similar values of $V_{th,SF}$. The average of the inter-HD among the devices with the basic post-processing was 64.0 bits (=50.0%), which was close to the ideal of 50%, indicating that the PUF ID of each device was unique. With the L.G. encoding, the average inter-HD shifted to around 60 bits and the uniqueness decreased, due to the invalid code in the Lehmer encoding.

It should be noted that the intra-HD and inter-HD were evaluated with 10-bit resolution, in which the lower 2 bits of a 12-bit digitized $V_{PUF}$ of the SF cell were decimated. This is because 12-bit line memory for image processing is reused for the PUF post-processing, as described in the following Section 4.1. Moreover, the average HD between 10-bit and 12-bit evaluation was only from 0.22% to 1.38% for the basic and L.G. post-processing as the lower 2 bits are less effective for the variation of $V_{th,SF}$.

Table 1 shows the comparison of the intra-HD and intra-HD among the presented CIS-PUF and other PUFs [22] without error correction circuits or physical variation enlargement.

The presented CIS-PUF shows comparable reliability and uniqueness to other PUFs. Next, the error rate is estimated through the use of probability theory.

### 3.2. Device Authentication Error Rate

**Figure 9:**
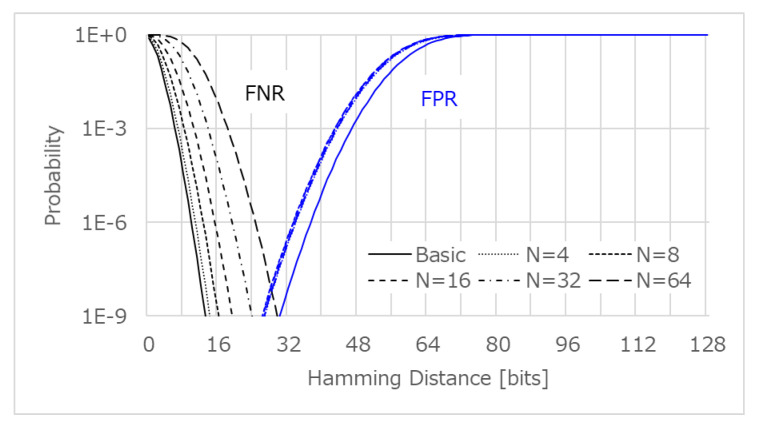
Estimated FNR and FPR estimated from the intra-HD and inter-HD.

Figure 9 shows the false negative rate (FNR) and false positive rate (FPR), estimated from the intra-HD and inter-HD.

In order to make the FNR less than $1\times 10^{-9}$ (=0.001 ppm) with the basic post-processing, an HD greater than 13 bits should be tolerated as random noise. On the other hand, FPR suggests that the HD should be less than 30 bits, in order to identify the enrolled device from mimic devices with an error rate of less than 0.001 ppm. Therefore, the device authentication will be successful as long as the verifier sets the threshold HD in the range of 13–30 bits. Similarly, the device authentication using L.G. post-processing will be successful, with an error rate less than 0.001 ppm, where n=4, 8, 16, or 32. However, the error rate is over 0.001 ppm with L.G. post-processing at n=64, for any threshold HD. Thus, we focused on the lightweight basic post-processing and the L.G. post-processing at n=32 for a larger number of device authentications, supposing that we need to identify a CIS device from a *trillion* CIS devices, which is expected in the IoT world.

## 4. Post-Processing Circuit Design

The advantage of the CIS PUF with device authentication is small circuit overhead. In this section, the circuit area for the post-processing is estimated.

### 4.1. Basic Post-Processing Circuit

**Figure 10:**
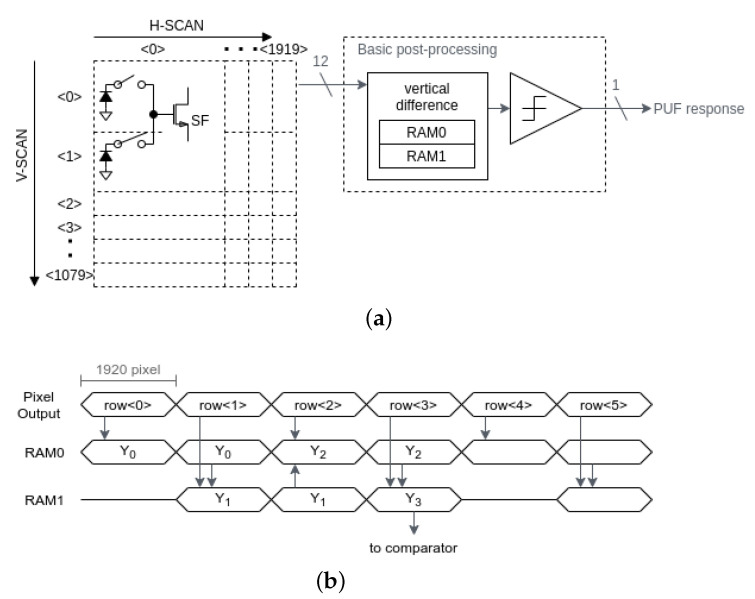
Basic post-processing circuit. (**a**) PUF response derivation circuit overview. (**b**) Timing diagram.

Figure 10 shows the basic post-processing circuit.

In the PUF mode, the SF cell array is read out with row-by-row scanning, in the same manner as the imaging mode. For example, the digitized $V_{th,SF}$ in the 0th row is first written in the 1920×12 bit line memory RAM0. As the CIS conventionally has 12 bit 1-line memory for image processing, the line memory is reused for the PUF processing. The lower 2 bits of each SF cell’s data are decimated, in order to avoid overflow of the 12 bit line memory, and this is given by

(7) $$\begin{array}{c} Y_{0}=\frac{row_{0}}{4}. \end{array}$$

The 1st row data are summed with the 0th row data in RAM0, and are then written into RAM1, as the same SF cell is accessed among this 2-shared pixel row scan. The operation is given by

(8) $$\begin{array}{c} Y_{1}=Y_{0}+\frac{row_{1}}{4}=\frac{row_{0}+row_{1}}{4}. \end{array}$$

The $V_{th,SF}$ in the second row data are subtracted from $Y_{1}$, in which the line signal $Y_{1}$ is moved back to RAM0 from RAM1 in order to avoid a further additional line memory. The operation is given by

(9) $$\begin{array}{ccc} Y_{2} & = & Y_{1}-\frac{row_{2}}{4}=\frac{row_{0}+row_{1}-row_{2}}{4}. \end{array}$$

The $V_{th,SF}$ in the third row data are subtracted from $Y_{2}$, in which the line signal $Y_{2}$ is again moved forward to RAM1 from RAM0. The operation is given by

(10) $$\begin{array}{ccc} Y_{3} & = & Y_{2}-\frac{row_{3}}{4}=\frac{row_{0}+row_{1}-row_{2}-row_{3}}{4}. \end{array}$$

Each vertical SF cell difference in $Y_{3}$ is sequentially binarized with the comparator. Thus, a 1920-bit PUF response is generated from a 1920×4 pixel array. The following pixel rows can be processed in the same way, in order to generate a 518.4-kbit PUF response. The PUF response is transmitted to the verifier in the timing sequence, similarly to the image data.

The circuit was designed with a Verilog HDL model and was synthesized using a 0.18 μm CMOS standard cell library [23], in order to provide an area estimate for the hardware implementation. As the image sensor input is an analog signal and CIS is often fabricated with a 65 nm to 0.18 μm CMOS image sensor process, the 0.18μm CMOS logic circuit is one candidate to realize low-cost CIS-PUF. The area was $0.65{\mathrm{mm}}^{2}$ or 50 k-gate, in which 97.5% of the area is dominated by the 2-line memories RAM0 and RAM1. The additional line memory to the conventional CIS is only the RAM1 because (1) the 4 line signal is processed with the 2 line memories by moving the halfway result back and forth among RAM0 and RAM1 and (2) the RAM0 is shared for image processing in the imaging mode and for the post-processing in the PUF mode. The area overhead to the conventional CIS was $0.33{\mathrm{mm}}^{2}$ or 26 k-gate, which is only around 1% of the total CIS chip area.

### 4.2. L.G. Post-Processing Circuit

**Figure 11:**
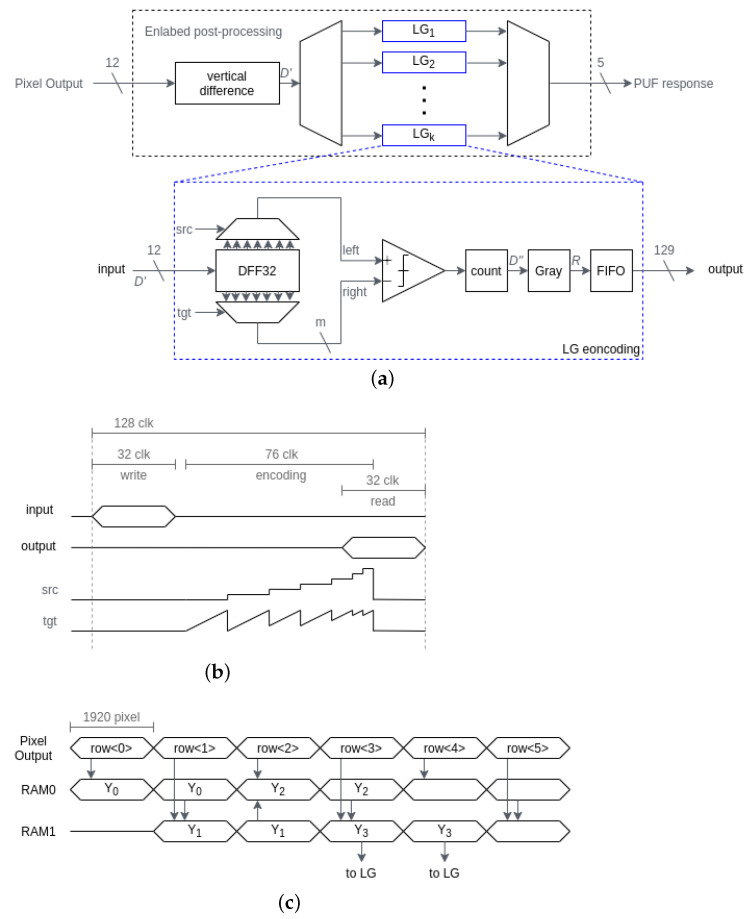
L.G. post-processing circuit. (**a**) Block diagram. (**b**) Timing diagram of LG encoding when m=8. (**c**) Timing diagram.

Figure 11 shows a diagram of the L.G. post-processing. The vertical difference circuit is the same as that in the basic post-processing circuit. The Lehmer encoding is time-interleaved among multiple modules, whose number is *k*, in order to process the sequential SF cell output. Each LG module stores 32 vertical differences, $D^{\prime }$, in data flip-flops DFF32. The Lehmer code counts the number of smaller terms of the *right*, which is smaller than the *left*, with a comparator and a counter, incrementing the value of *tgt*, which is the *right* selector input. The value of *src*, which is the *left* selector input, is then incremented and the number of smaller terms of the *right* than the shifted *left* is counted. The counted Lehmer code $D^{\prime \prime }$ is Gray-encoded and the PUF response *R* is output in a first-in first-out (FIFO) manner. The clock period for the counting depends on the number *m*, in which *m* bits of the *right* signal are simultaneously compared to the *left* signal in parallel. If m=1, the counting period is 496(=31·32/2) clock. As *m* increases, the period decreases, while the area of the LG encoder increases for parallel processing. If m=8, the counting period is 76 clock, and the total period for the L.G. encoding is 128 clock, as shown in Figure 11b. Therefore, the number of time-interleaving LG modules is 4(=128/32). Moreover, a 2-row period was assigned for 1-row data processing, as shown in Figure 11c. In particular, a half of the vertical difference $D^{\prime }$ in $Y_{3}$ is Lehmer and Gray-encoded during the readout of the SF cell in $row_{3}$, and others are encoded in the background of the readout of the SF cell in $row_{4}$. The number of LG module is, thus, halved to be 2.

In order to minimize the area overhead to the conventional CIS, (1) FIFO manner, (2) simultaneous comparison (m=8), and (3) 2-row period processing are presented. Even though the number of time-interleaving circuits is 18 when these techniques are not utilized, the number of time-interleaving circuits is reduced to only 2 with the three techniques. The synthesized area was therefore only $0.90{\mathrm{mm}}^{2}$ or 69 k-gate, of which 71.2% of the area was dominated by the 2-line memories RAM0 and RAM1. The area overhead was $0.57{\mathrm{mm}}^{2}$ or 44 k-gate, around 2% of the total CIS chip area.

### 4.3. Circuit Area and PUF Response Length

The basic post-processing provides a more lightweight security function, and L.G. post-processing is utilized when a larger number of device authentications is required. Table 2 summarizes the characteristics of the basic and the L.G. post-processing circuits. The area overhead of the basic and L.G. post-processing circuits are 26 k-gate and 44 k-gate logic, respectively. Though the area overhead of L.G. post-processing is 69% larger than that of the basic post-processing, the effective number of PUF response bits in the L.G. post-processing was 3.7 times larger than that of the basic post-processing. The LG post-processing is more area-efficient for a PUF response bit.

It is noted that, if 518 kbit SRAM is implemented as the source of the PUF ID in the CIS, the area overhead will be 20 times larger than the CIS-PUF with the basic post-processing. If 1902 kbit SRAM is implemented as the source of the PUF ID in the CIS, the area overhead will be 45 times larger than the CIS-PUF with the L.G. post-processing. The CIS-PUF is area-efficient to realize the image information security that starts from the CIS, because the pixel transistor and some circuits are used for the imaging mode and the PUF mode.

## 5. Summary

In order to realize image information security starting from the data source, two PUF post-processing modes that are resistant to modeling attacks for CR device authentication with a 2-Mpixel CIS were studied. The basic post-processing generates the PUF response, reducing the column FPN to improve the uniqueness of the PUF ID. For the 2-Mpixel CIS with basic post-processing, the total number of PUF responses is 518.4 kbit and 4050 device authentications can be carried out with an error rate less than 0.001 ppm. If a larger number of device authentications is required, the L.G. post-processing can be utilized to carry out authentication 14,858 times. The area overhead for the CIS chip with the basic post-processing and the L.G. post-processing was only 26 and 44 k-gates, respectively, based on a Verilog HDL model circuit design. As the area penalty is only 1% or 2% of the total CIS chip area, this technology can serve to realize low-cost image information security.

## Figures and Tables

**Table 1:** Comparison table of intra-HD and inter-HD.

PUF	CIS	CIS (L.G.)	SRAM	Latch	DFF	RO	RO (L.G.)
		n=16					n=16
$\mu_{intra}$	1.24%	2.96%	5.46%	2.86%	3.56%	1.53%	3.56%
$\sigma_{intra}$	0.98%	1.54%	0.14%	0.28%	0.31%	0.39%	0.63%
$\mu_{inter}$	50.0%	47.2%	49.7%	35.0%	42.0%	49.6%	46.9%
$\sigma_{inter}$	4.42%	4.49%	0.32%	1.52%	0.90%	1.11%	0.48%

**Table 2:** PUF response and circuit area.

	Area Overhead [Gate]	#PUF Response [Bit]	Efficiency [Gate/Bit]
Basic	26 k	518 k	0.050
L.G.	44 k	1902 k	0.023
